# The Amber-Colored Liquid: A Review on the Color Standards, Methods of Detection, Issues and Recommendations

**DOI:** 10.3390/s21206866

**Published:** 2021-10-16

**Authors:** Muhamad Haziq Hasnul Hadi, Pin Jern Ker, Vimal A. Thiviyanathan, Shirley Gee Hoon Tang, Yang Sing Leong, Hui Jing Lee, Mahammad A. Hannan, Md. Zaini Jamaludin, Mohd Adzir Mahdi

**Affiliations:** 1 Institute of Sustainable Energy, Universiti Tenaga Nasional, Kajang 43000, Malaysia; se23032@utn.edu.my (M.H.H.H.); angela@uniten.edu.my (V.A.T.); hannan@uniten.edu.my (M.A.H.); 2 Department of Microbiology, Faculty of Medicine, Manipal University College Malaysia, Bukit Baru, Melaka 75150, Malaysia; shirley.tang@manipal.edu.my; 3 International Medical School, Management and Science University, Shah Alam 40100, Malaysia; 4 Department of Electrical, Electronic and System Engineering, Faculty of Engineering and Built Environmental, Universiti Kebangsaan Malaysia (UKM), Bangi 43600, Malaysia; p107849@siswa.ukm.edu.my; 5 Institute of Power Engineering, Universiti Tenaga Nasional, Kajang 43000, Malaysia; LHjing@uniten.edu.my (H.J.L.); mdzaini@uniten.edu.my (M.Z.J.); 6 Wireless and Photonics Networks Research Centre, Faculty of Engineering, Universiti Putra Malaysia (UPM), Serdang 43400, Malaysia; mam@upm.edu.my

**Keywords:** amber, color, detection, liquid, color measurement

## Abstract

For most natural or naturally-derived liquid products, their color reflects on their quality and occasionally affects customer preferences. To date, there are a few subjective and objective methods for color measurement which are currently utilized by various industries. Researchers are also improving these methods and inventing new methods, as color is proven to have the ability to provide various information on the condition and quality of the liquid. However, a review on the methods, especially for amber-colored liquid, has not been conducted yet. This paper presents a comprehensive review on the subjective and objective methods for color measurement of amber-colored liquids. The pros and cons of the measurement methods, the effects of the color on customer preferences, and the international industry standards on color measurements are reviewed and discussed. In addition, this study elaborates on the issues and challenges related to the color measurement techniques as well as recommendations for future research. This review demonstrates that the existing color measurement technique can determine the color according to the standards and color scales. However, the efforts toward minimizing the complexity of the hardware while maximizing the signal processing through advanced computation are still lacking. Therefore, through this critical review, this review can hopefully intensify the efforts toward finding an optimized method or technique for color measurement of liquids and thus expedite the development of a portable device that can measure color accurately.

## 1. Introduction

Color is a subjective perception and interpretation of what an individual sees and observes. Defining what “color” really means can be challenging [1,2]. Color is a visual sensation attribute, and the color appearance of an object is determined by three factors: the light source, object and visual system. An object appears to have colors because it absorbs certain optical wavelengths coming from the light source and reflects certain wavelengths. An object that can produce a spectrum at a particular wavelength or a certain waveband, such as a flame or laser, is known as a self-luminous object. The emitted spectrum of the self-luminous object affects the spectrum reflected from the illuminated sample, resulting in differences in color determination. Regardless of how difficult it is to define color, the various attributes of color can be defined much more precisely, and these are the terms that are the most important in color appearance modeling. Typically, color is expressed using three dimensions which include, hue, chroma, and lightness [3,4]. Hue is how an individual recognizes an object’s color (e.g., blue, yellow, green, or red). Chroma shows how close the color is to gray or a pure hue, while lightness represents the comparison of the color as light or dark. However, those three dimensions are insufficient to fully specify color appearance. In fact, a complete specification of color appearance requires five perceptual dimensions: hue, chroma, colorfulness, lightness, and brightness [1,2].

In various industries, color is an important attribute related to the quality of a product, as it influences businesses and consumers’ choices and preferences. Generally, the color of the material or product is the first parameter that consumers perceive which can be used for acceptance or rejection of the product by manufacturers and producers [5,6]. Therefore, color is often used for quality control in many fields, such as food industries [4,7], agriculture [8], dentistry [9], forensics [10], oil-based liquids [11,12] and water [13,14]. It is also undeniable that color reflects the condition, chemical content [15,16,17] and properties of materials and products [5]. The color variation is mostly dependent on the origin of its source, methods of production [18,19,20] and standard procedure of storage [21,22]. In addition, changes in color may be indicative of problems in the production process, contamination, degradation or the oxidation of the materials and products [23,24]. The manufacturers and producers can then carry out the necessary actions in response to the change of color in the materials and products. Hence, it is important to have a systematic color measurement technique, procedure, color scales and equipment to ensure the color is appropriately described.

Currently, the conventional methods of determining the color are via visual inspection [25] and by using a color comparator [26,27]. However, these methods are considered subjective as they involve human observation, which may introduce errors during measurement. Therefore, the objective of this paper is to provide a review of recent developments in color measurement for oil products and other amber-colored liquids. For mineral oil, its quality depends on its properties, such as the viscosity [28], breakdown voltage [29], inhibitor content [30], color [11,31], acidity [17] and moisture content [23]. Depending on its applications, different chemical and additives are added, which results in shades of colors. Changes in color for mineral oil are mainly due to degradation. Oxidative stress occurs on the mineral oil with the presence of oxygen, moisture-producing polar compounds and oil sludge, which causes the mineral oil to degrade. Other than that, degradation of mineral oil is also caused by continuous stresses such as heat, electrical [32], and mechanical stress. Therefore, color identification can provide an early indication of the condition of mineral oil for immediate remedial action, thus reducing the cost for maintenance and changing the oil [32]. Once the condition of the mineral oil is known, the next maintenance step will be taken.

As for common consumer products with amber-colored liquids such as cooking oil, honey, maple syrup and beer, consumers evaluate the color of the product apart from the product packaging, price and label information [33]. The color of these amber-colored liquid products is highly related to its content, flavor and taste. For example, certain major vegetable oil-producing countries prefer a certain color of oil because of its correlation with the flavor [34]. Other than that, the color of the honey and maple syrup also plays an important role in the market price of the products [35]. Appropriate color measurement allows exporters and producers to choose the most beneficial trading market for their products [33,36]. However, general consumer preferences toward the color of amber-colored liquid are still inconclusive.

Generally, different types of amber-colored liquid have their own methods for color determination. There are fixed standard procedures which need to be adhered to when conducting the color measurement. Research was also carried out to identify the best method for color determination of liquids that are suitable with the current technology. Furthermore, there are also many established international standards that provide a detailed procedure with the necessary apparatus and materials for color measurement [25,27,37,38]. This demonstrates the importance of color determination of liquids in many industries and fields.

Despite the importance of color determination, there is currently no comprehensive review on the methods or techniques for the color determination of amber-colored liquids, although there are a few reviews reported on the color determination for food [4,7], dental [9] and oil-based liquids [5,32]. Therefore, this review will emphasize various color standards and methods for color determination of amber-colored liquids, such as mineral oil, vegetable oil, honey, maple syrup and beer. A comprehensive and thorough discussion on the conventional and objective methods of color determination for these liquids has been presented. The advantages along with the issues and challenges related to the conventional and objective methods, as well as the recommendations for research opportunities, have been thoroughly discussed and presented. This is to promote the utilization of a more practical and effective color determination method based on advancement in sensing technologies and signal processing.

## 2. Review Methodology

The preparation of this review paper can be divided into two phases. The first phase is the initiation of a research theme to create appropriate research questions and develop the objectives of the manuscript. The second phase involves a comprehensive and unbiased literature search of academic articles (e.g., proceedings, books, journals and official websites) using Google Scholar, and the literature examples are organized accordingly based on the research questions. A total of 150 articles were collected, and after filtration, the 105 most relevant articles were chosen and added to the review paper. In addition, about 95% of the resources cited in this paper were published in the years 2001–2020. Some of the essential keywords used in the literature search included oil color, color determination of liquids, oil color measurement and methods for color determination of liquid.

## 3. International Standards for Color Determination

One of the methods for color determination is by using the human eye. The person who is conducting the observation must use their perception and experience to describe the color of the sample. Although this method may be fast, it produces inconsistent results, because a different person may report a different color depending on how they perceive the color of the sample. This leads to the introduction of standardized color scales for more consistent results. These established color scales contain a palette of available colors to reproduce or grade the color of the samples in certain values. Different color scales have different values, depending on the standard that is established for different samples. The grading technique is often used to assess a sample’s color by comparison with a representative series of fixed color standards.

The established standards will provide the color scale and the standard procedure of conducting the test methods to measure the color of the liquid. The standards also recommend a specific experimental set-up or measuring device to be used for the measurement [27,39]. Different liquids and oils have different color standards that have been established by authorized standard bodies. In this section, the international standards related to the color determination of amber-colored liquid such as transformer oil, lubricant oil, olive oil, palm oil, honey and beer will be fully described. The equipment used and the methodology of measurement will be discussed and elaborated.

### 3.1. CIE Values

The Commission Internationale de I’Eclairage (CIE) is a non-profit international organization that regulates standards for all aspects of lighting and illumination, comprising colorimetry and photometry. The CIE color system consists of three color coordinates to identify a color in the color space [3]. The color spaces include CIE *XYZ*, CIE L*a*b* and CIE L*C*h coordinates [40]. Color can be measured and calculated using the color spaces based on the specification of the light source and observers with constant evaluation.

**Figure 1:**
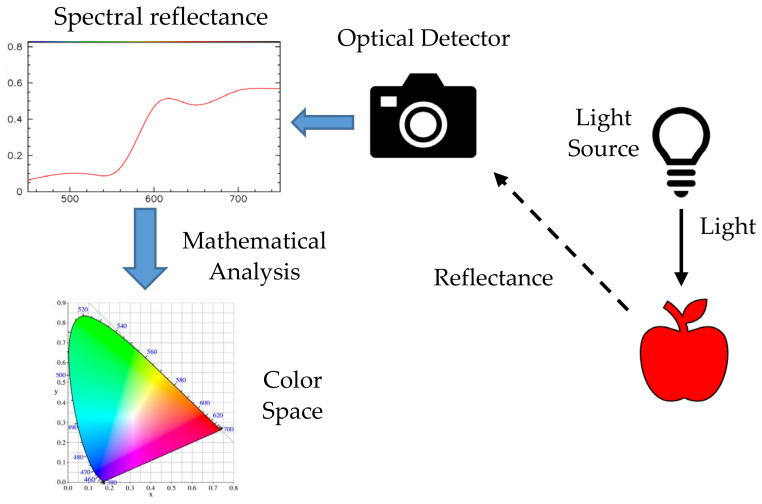
Color detection process.

Color, according to the definition by the CIE, is the characteristic of visual perception that can be described by the attributes of hue, brightness or lightness and colorfulness, saturation or chroma [41]. Color is measured by the wavelengths of light reflected or transmitted from an object. The instrument containing an optical detector records the intensities of different optical wavelengths as points across the visible spectrum. Once the spectral reflectance curve is obtained, mathematical analysis is applied to map the color onto the color space. Figure 1 shows the example of a basic process for color detection from the interaction of light, the object, the detector and the signal processor.

To identify the color numerically, the concept of a standard observer that is based on the average human response to wavelengths of light is derived. A standard observer represents how an ordinary person sees color across the visible spectrum when using a defined area of the eye’s retina.

**Figure 2:**
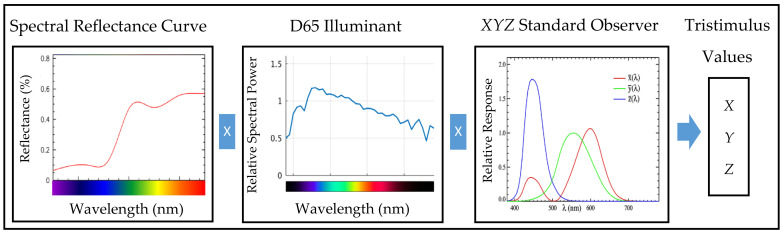
Tristimulus values.

CIE *XYZ* color spaces are also known as tristimulus values. These are measured with the standard illuminant, the spectral curve of the sample and a standard observer as shown in Figure 2. *XYZ* are extrapolations of red, green and blue (RGB) color models that are created mathematically to avoid negative values. The negative values were removed to match all physically realizable color stimuli. The general equations for *XYZ* are given below [1,2,42]:

(1) $$X=k\int_{\lambda }\Phi \left(\lambda \right)\bar{x}\left(\lambda \right)\;d\lambda$$

(2) $$Y=k\int_{\lambda }\Phi \left(\lambda \right)\bar{y}\left(\lambda \right)\;d\lambda$$

(3) $$Z=k\int_{\lambda }\Phi \left(\lambda \right)\bar{z}\left(\lambda \right)\;d\lambda$$

where Φ(λ) is the spectral power distribution of the stimulus, $\bar{x}\left(\lambda \right)$, $\bar{y}\left(\lambda \right)$ and $z\left(\lambda \right)$ are the color-matching functions, *k* is the normalizing constant, *X* is a mix of the cone response curves chosen to be orthogonal to luminance and non-negative, *Y* is the luminance and *Z* is in the blue area. The chromaticity and color space in Figure 1 were derived from the *XYZ* color spaces. The *xy* coordinates were used to form the chromaticity. The notation *Yxy* specifies colors by identifying the lightness (*Y*) and the color as viewed in the chromaticity diagram (*x*,*y*).

CIE L*a*b* is a color space that can be considered a color appearance model. This color space includes simple chromatic adaptation transformations and predictors of lightness, chroma and hue [2]. The L* is the brightness, a* denotes the red and green value, and b* is the yellow and blue value. The CIE L*a*b* color space contains all perceivable colors, and its color spectrum exceeds the color models of RGB and cyan, magenta, yellow and black (CMYK). L*a*b* is not RGB or CMYK, but a different color model. It is a purely theoretical color space that is sometimes used as an absolute standard for comparing all other color spaces. The color plotting diagrams for L*a*b* can be found in [3,40]. A movement in the +a* direction of color measurement represents a shift toward red. Movement of +b* along the b* axis indicates a change to yellow. The middle L* axis displays L* = 0 at the bottom (black) and L* = 100 (or white) at the top. In between L* = 0 and L* = 100 are gray values. All colors can be regarded as neutrals on this axis, as they are not colored in any direction.

While CIE L*a*b* uses Cartesian coordinates to measure color in a color space, polar coordinates are used by CIE L*C*h. In other words, the CIE L*C*h color space is a vector representation of the CIE L*a*b* color space [2]. The *L* defines the lightness, *C* specifies the chroma and *h* denotes the hue angle, an angular measurement. CIE L*C*h uses cylindrical coordinates instead of Cartesian coordinates. Delta E ($\Delta E_{ab}{}^{*}$) is the total color difference between two stimuli that is based on the difference of L*, difference of a* and difference of b*. *DE* is measured by a scale from 0 to 100, where a bigger value indicates distinction between two colors.

### 3.2. Lovibond RYBN

The Lovibond red, yellow, blue and neutral (RYBN) color scale is designed for the color measurement of transparent but colored liquids. The color scale is widely used for beer, malts, caramels, edible oils and fats, food and beverages, petroleum oils and waxes [3,26,43]. The color scale is based on 84 calibrated colored glass standards of red, yellow, blue and neutral glasses with various densities ranging from unsaturated to fully saturated [3,39]. Each color of RYBN has its own unit range. The color of the sample is matched by the closest combination of the three primary colors along with neutral filters to form a set of Lovibond RYBN units that defines the color. Table 1 shows the RYBN color range. Since there are several million combinations available, most samples will match their colors with this color scale.

Lovibond has set a guideline on how to operate the measurement using the RYBN color scale. However, alterations to the procedure during the measurement can be made for any reason. The operators who conduct the measurement must provide detailed information on the whole process to prevent other operators’ ambiguity. As an example, in the case of employing neutral filters to dull a bright sample, the operator failed to report the process. A sample is described as bright when the nearest possible match appears dull in comparison. When this occurs, neutral values are added and recorded as the sample brightness. In other cases, the operator tries to make the best possible match without using neutral values—although they are necessary to use—or randomly use a different color combination in a fixed ratio.

The Lovibond RYBN colorimeter is made up of two adjacent fields of view. When conducting the measurement, the operator needs to see through the viewing tube. The operator needs to make sure that the sample in the sample field is observed side by side with a white reflective surface in the comparison field. Both the sample and the comparison field must be suitably illuminated. Lovibond RYBN standard glasses are also used for comparison with the color of the light. The light can either be transmitted through or reflected from the sample [3]. A series of neutral glasses are used in the sample field to “dull” the color of the sample, which is too bright to obtain a good color match using red, yellow or blue glasses. A sample is reported as “bright” when the nearest possible color match appears dull in comparison. Neutral glass values are introduced and recorded as the sample brightness.

### 3.3. ASTM Scales

The American Society for Testing and Materials (ASTM) is an international standard body that develops ASTM standards and procedures to standardize the analysis of the physical, mechanical, rheological, thermal and chemical properties of many types of materials, products, systems and services [44]. Other than that, ASTM also introduces color standards and color scales to have a standardized color measurement. These standards are developed specifically for color determination of a spectrum of petroleum oil products such as lubricating oils, heating oils, diesel fuel oils and petroleum waxes [45]. The determination of color for petroleum products is mainly for industrial purposes. It is an important parameter to be analyzed, since color can give the first indication of the quality of the oil.

#### 3.3.1. ASTM D 1500

**Figure 3:**
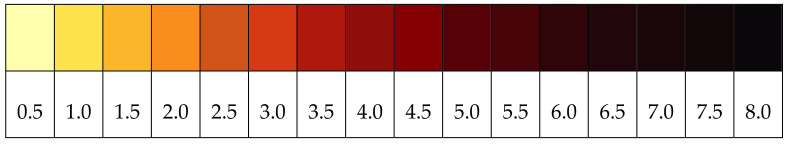
ASTM D 1500 Color Scale.

The ASTM D 1500 is a standard test method for ASTM color of petroleum products. It introduces ASTM color scale containing 16 ASTM color indices ranging from 0.5 for the lightest color to 8.0 for the darkest color, with steps of 0.5 [3,27] as shown in Figure 3. A color comparator is recommended as the tool for color measurement of the oil sample. By using a standard light source with color temperature of 2750 K, oil sample in a standard glass jar is placed in the instrument, and it is compared with two colored glass disks, consisting of the 16 ASTM color.

#### 3.3.2. Saybolt Color

Saybolt chromometer method is adopted as a standard test by ASTM D 156, United States Fuel Association, National Petroleum Association, and American Petroleum Institute. Other than product quality, the color scale is often used to determine the overall purity of fuels such as kerosene, jet fuel, gasoline and naphtha [27].

**Figure 4:**
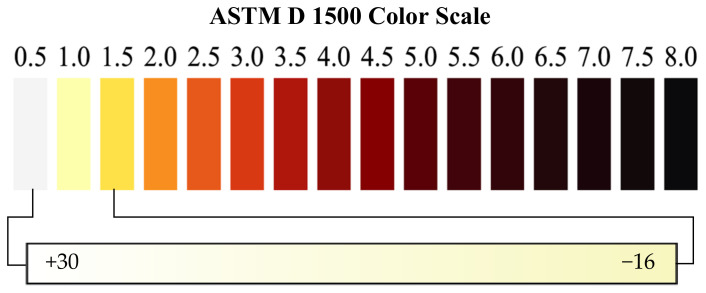
Saybolt color scale with ASTM D 1500 color scale.

The color standard varies from near water white (30) to dark yellow (−16). Comparing to the ASTM D1500, this color scale is recommended for samples with lighter color. Figure 4 shows the differences between Saybolt Color and ASTM D 1500 color scale.

#### 3.3.3. Gardner Color

The ASTM D 1544 and ASTM D 6166 are standards that use the Gardner color scale. The Gardner color scale is a single number with a series of 18 color variations ranging from near clear, light yellow (Gardner 1) to dark brown (Gardner 18) [3,27]. Gardner 0 is defined by clear distilled water. The scale initially consisted of 18 liquid standards sealed in glass tubes for visual comparison. The chromaticity of the liquid standards in glass tubes defines the color scale. Apart from oil, this color scale is also used for paints, and chemicals such as resins, varnishes, lacquers, fatty acids, and lecithin. 

According to the standards, the procedures for the measurement involves comparing the sample with the liquid standard. The liquid standard that matches the color of the sample closely is determined in terms of brightness and saturation. Other than that, a Gardner color comparator can also be used with a 3-section field of view. The sample and two adjacent color standards are viewed and measured simultaneously, making it easier to achieve the most closely-matched color.

### 3.4. Pfund Scale

The United States Department of Agriculture (USDA) categorizes honey into seven colors: water white, extra white, white, extra light amber, light amber, amber and dark amber [3,39,46]. The Pfund color grader is a measuring device that is used primarily by the honey industry. This color scale provides continuous readings for the entire range of colors of honey. The Pfund color grader visually compares a standard amber-colored glass wedge with a honey sample contained in a wedge-shaped cell. The color intensity of the honey is expressed in millimeters (mm) along with the amber wedge, and the color usually ranges from 1 mm to 140 mm. Table 2 shows the Pfund color scale in mm with its color name used for color measurement of the honey. The mm unit is the distance the wedge must be moved for the color of the sample to match the color scale. The Pfund color grader is affordable and easy to use, but readings can differ from instrument to instrument due to scale limitations. Measurements using the Pfund color comparator have less sensitivity to detect a slight difference between samples, which can lead to misleading results compared with Pfund values derived from spectroscopy measurements [47].

### 3.5. Beer Color Scale

The standard method for the color determination of beer in the United States is the Standard Research Method (SRM) introduced by the American Society of Brewing Chemists (ASBC) [49]. Initially, this technique was set up to approximate according to the Lovibond RYBN scale. However, the ASBC has identified a method of color determination that can provide good correlation with the visual methods. Thus, the ASBC recommended a method based on spectrophotometer readings for the color determination of beer, which is named the SRM. The SRM scale is primarily used in North and South America.

This method utilizes a spectrophotometer to measure the amount of light absorbed by the beer in a cuvette when it is illuminated with white light. Although a spectrophotometer with white light is used, the SRM only utilizes the absorbance value at a wavelength of 430 nm. The SRM color value can be obtained by applying Equations (4) and (5):

SRM = A_430_ × 10 (4)

SRM = A_430_ × 12.7 (5)

where SRM is the SRM value and A_430_ is the absorbance measured at a wavelength of 430 nm.

A beer sample is measured in a cuvette with a path length of 1.27 cm. The resultant absorbance value is then multiplied by 10 as in Equation (4) to yield the color value and by any dilution factor if the sample is diluted to bring the color within a reliable measurement range. If a 1-cm path length cuvette is used, then a multiplier of 12.7 is used instead as shown in Equation (5). The multiplier is changeable according to the path length of the cuvette used.

Similarly, the European Brewing Convention (EBC) color standard was developed by the Institute of Brewing and the European Brewing Convention. It is a method for color grading beers as well as similarly colored liquids [49]. Initially, beer color was estimated qualitatively by comparing colored glass references based on the Lovibond RYBN scale to the beer samples. This EBC color standard is commonly used in Europe.

For the EBC method, the measurement of beer color is conducted in the same fashion as the SRM by reading the absorbance of light at 430 nm but in a cuvette with a path length of 1 cm. The EBC value can be calculated by using Equation (6):

EBC = A_430_ (1 cm cell) × 25 (6)

The EBC method previously measured the absorbance at a wavelength of 530 nm. The European industry later changed and adopted the absorbance at a wavelength of 430 nm so that the final measured color was according to the Lovibond RYBN references. Therefore, for the same color of beer, EBC units are approximately twice as large as SRM color units. 

Table 3 below shows a comparison of the beer color units between the SRM and EBC color scales.

### 3.6. Maple Syrup Color Scale

In Canada, there are two types of maple syrup color classification: Canadian federal and Quebec provincial classifications [50]. The Canadian federal classification is made up of three classes and five grades of color: Canada 1, (extra light, light and medium), Canada 2 (amber) and Canada 3 (dark). The classes and color grades are as shown in Table 4.

The Quebec provincial classification is made up of two classes and five grades: AA (extra light), A (light), B (medium), C (amber) and D (dark amber). The classes and color grades are as shown in Table 5.

For the United States of America, the characterization of maple syrup is divided into three grades by color and flavor under the authorization of the USDA [37]. According to the authorized bodies for maple syrup classification of both countries, the color is classified based on the intensity of visible light transmitted through the syrup by spectrophotometer measurement at a wavelength of 560 nm [37]. Table 6 shows the “Grade A” maple syrup color that was then agreed upon by both Canada and the United States of America to have the same standard for the color grading system [37].

The “processing grade” classification is for any maple syrup that fails to meet Grade A criteria but has a relatively good maple taste, good visualization and can contain off-flavors. Any maple syrup that fails to meet the criteria of these two grades is placed under the substandard classification.

To summarize Section 3 on the international standards, Table 7 shows all the color standards which are commonly used in color determination of amber-colored liquids. The color range of the color scales is determined by the authorized bodies based on the applications and the liquids to be measured. The methods and procedures also vary in the measurement of amber-colored liquids based on different color standards.

## 4. Methods for Color Measurement

The methods of determining the color of liquids can be divided into two groups: subjective methods and objective methods. For subjective methods, human interaction and judgment are involved during measurement, while for objective methods, the instrument will conduct the measurement with no human judgment involved. Therefore, a significant difference in measurement between these two methods can be observed. In this section, both subjective methods and objective methods, which were studied by researchers with various types of amber-colored liquids, will be discussed in detail. The methodology, including the color standard used, equipment and findings of the previous work, will be reviewed.

### 4.1. Subjective Methods

Subjective methods depend largely on how a person interprets a certain color based on historical experience or with the aid of color references. In this subsection, subjective methods that were used commonly in the industry or are being investigated by researchers will be discussed.

#### 4.1.1. Visual Examination

The most straightforward method of color determination is through a visual examination where human sight is used [25]. Trained or experienced personnel are required to conduct the examination to obtain reliable results. The color of a sample in a glass jar will be compared with another sample that is used as a reference [52]. No special analytical equipment or chemicals are required for the color measurement. For example, Nagao et al. (2012) determined the colors of different types of cooking oil, which consisted of soybean oil, blended canola and soybean oil and canola oil with or without lard obtained from various places [52]. The cooking oil samples were placed in a Gardner-Holdt tube or any similar shape container. They were then compared side by side with a colored liquid standard. Although this method can give fast results and can be conducted on site, the color reported by one person can be different from that of another person, as a different person may perceive the colors with slight differences. For example, a person may perceive a sample color of 4 Gardner Index as light yellowish, but another person may see it as pale yellow, which might be lower than 4 Gardner Index. The decision making for this method is theoretical and merely based on personal assumptions and one’s sensory ability. The lighting conditions are also a crucial factor in conducting visual examinations. Therefore, the accuracy of the color identified is ambiguous and not consistent. During the visual examination of oils, color, transparency and foreign objects are examined to decide whether further analysis is needed.

#### 4.1.2. Visual Color Comparator

A visual color comparator is an instrument used to measure the colors of the samples by comparing them with a few standard colored discs [26]. One example of a color comparator is the Lovibond Comparator 3000, which utilizes this concept for measuring the color of a sample.

Most of the organizations or institutions that establish the standards and color scales for color measurement have a color comparator to ensure that the measurement is standardized. The standards that use this instrument are the ASTM color scale and the EBC. Each standard has its comparator disc and technical data. For example, for conventional measurement using the ASTM color scale, if the color of the sample matches any of the color discs, the color is reported. Otherwise, if the sample color is between two standard ASTM colors, the darker glass of the ASTM color preceded by the letter “L” is reported [27]. In many cases, the measured sample does not fall exactly on the values in the color scale. Thus, the exact color of the sample is not precisely identified through this method.

#### 4.1.3. Visual Colorimeter

A visual colorimeter is a measuring device that measures the color of a sample using combinations of 84 colored glasses, expressed in terms of red, yellow, blue and neutral. Each colored glass has a scale of tens, units and decimals. The light source used for this device is approximately the same as the daylight color temperature, which is about 5000–6500 K. The color of the sample is then measured by observation through the viewing tube. Then, the sample in the field and a white reflective surface in the comparison field are observed side by side. Therefore, both the sample and the white reflective surface are suitably illuminated. The operator needs to adjust the colored glass racks until a visible color match is identified for the light from the sample, and its color can then be expressed in Lovibond RYBN units. Different international standard bodies may have different formats of color combinations that meet the requirement of their official methods for color measurement. Most of the vegetable oils are measured for their colors using the visual colorimeter. A few examples are provided in the following sections.

i. Palm Oil

The color of crude palm oil (CPO) was first measured using the Lovibond colorimeter. Then, the color and quality prediction of refined, bleached and deodorized (RBD) palm oil were studied using an artificial neural network (ANN), where the final quality prediction was observed [53]. In the ANN model, three multiple-input single-output (MISO) and two multiple-input multiple-output (MIMO) algorithms were developed. Overall, the findings showed that all networks gave better regression performance, which had a correlation accuracy of more than 80% with the actual process parameters, except for MISO-1, which yielded a correlation accuracy of less than 60%. This was due to the distribution of data, which significantly affected the performance. However, when comparing single-output networks with multiple-output networks, multiple-output networks gave superior predictions of the output, especially MIMO-2. Table 8 shows the ANN model along with the output prediction and the regression (R) and mean squared error (MSE) values.

ii. Chia Seed Oil

Though there is still no report on the standard measurement of chia seed oil color, the most commonly used method is colorimetry. Therefore, color scales such as CIE L*a*b* and Lovibond RYBN are used for color measurement. The color measurement of chia seed oil provides information on the effect of the extraction method on the physicochemical properties and quality characteristics of the oil [18,54], as well as the origin of the chia seed [55]. Other than that, measurement with a conventional Lovibond Tintometer is used, where the color is expressed in red (R) and yellow (Y) value units [55,56,57]. A 13-cm quartz cuvette was used by Imran et al. (2016) for measurement so that the color changes of the sample could be observed clearly. Changes in the R and Y value units can also be used as an indicator for distinguishing the degree of metamorphism of chia seed oil.

iii. Mustard Seed Oil

Colorimeter is still commonly used to measure the color of mustard seed oil [58,59]. Bijay Krishna et al. (2009) measured the color of bleached mustard oil using a Lovibond Tintometer. In another study, Nayak et al. (2016) investigated the quality parameters of mustard oil after being heated for 30 h. Correlation between the physical and chemical properties of the oil was studied, where the color was expressed in CIE L*a*b* values [59].

#### 4.1.4. Issues and Challenges

Although these subjective methods are still commonly used by certain industries, some issues can be improved and overcome. One of the major issues related to the subjective method is the involvement of human interaction and observation. The reported color can be influenced by their judgment based on previous experiences of conducting color measurements. Furthermore, the use of a colorimeter also involves human interaction. Although some colorimeters like the Lovibond Tintometer are in accordance with a standard, when human judgment is involved, the outcome can be varied and inconsistent. This is due to different persons perceiving colors differently. Chromatic adaptation also affects the colors of objects perceived by humans. Without having fully chromatic adaption for more than 1 min to reach a steady state, the observer’s judgement on color will possibly be different and incomparable [60]. Although this method can measure the color of the sample with a fast result, the result obtained may not be consistent and accurate. This inconsistency may also be affected by the quality of the light source used in the colorimeter, thus affecting the accuracy of the measurement.

Apart from that, the number of measurements conducted per day using this method is limited. Long hours of exposure to a high intensity of light leads to eye problems and headaches. The operator is required to rest their eyes after measuring each sample according to the occupational safety and health guidelines. The operator also needs to perform eye checks frequently for health and safety reasons. Furthermore, the operator needs to be trained first before conducting any measurements so that the results obtained are in accordance with the sample measured. This limitation can be addressed using objective methods, an area of study which is beneficial.

### 4.2. Objective Methods

The standardized and high accuracy of objective methods has gained the attention of many researchers as an alternative to subjective methods. In this subsection, objective methods such as an automatic color comparator, automatic colorimeter, optical spectroscopy and image analysis that was used commercially in the industry or being investigated by researchers on amber-colored liquid will be discussed.

#### 4.2.1. Automatic Color Comparator

The development of an automatic color comparator device eliminates the dependency on physical color differences and human judgment for color determination. It increases the speed and accuracy of color determination for a sample. The automatic color comparator compares the color of the sample with the color that has been installed in the device by measuring the sample light absorbance using a white light-emitting diode (LED) as the light source and tristimulus detectors to detect the intensity of the light. The operators observe the color difference with the on-screen color or on-screen numerical display on the device. Once the color has been identified, it will display on the screen the color index of the sample according to a particular color standard. The standards that use this instrument are ASTM color, Gardner color and Saybolt color. Each of the standards will have its specific comparator setting and technical data.

#### 4.2.2. Photoelectric Colorimeter

**Figure 5:**
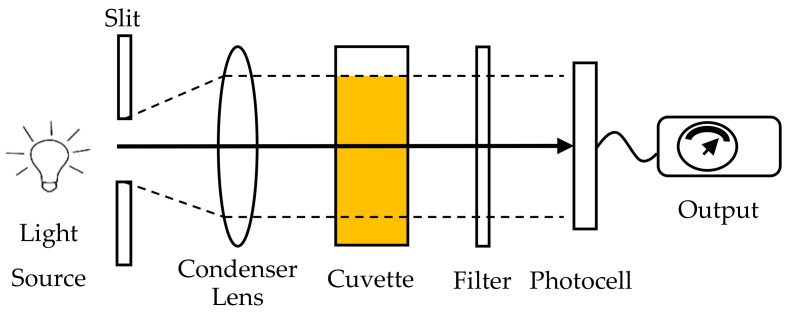
Photoelectric colorimeter diagram.

Measurement using a visual colorimeter is slow and tedious because it needs an operator to manually operate the device to measure color and uses his or her own perception in determining the color of the sample. However, a photoelectric colorimeter was introduced, which could reduce the time for the measurement and increase the reproducibility of the measurement as it uses a photocell, a set of color filters, an amplifier and an indicating meter for the quantitative determination of color. Colorimetry is a scientific technique for the measurement and classification of color that replaced the subjective method with an objective numerical system. A colorimeter is a measuring device for measuring the transmittance or absorbance of light after passing through a sample [61]. The three main parts of a colorimeter are a light source, a sample holder to hold the sample solution and a photocell that detects the light being transmitted through the sample. Figure 5 shows the diagram of a photoelectric colorimeter. Commonly, the light source used in the device is a tungsten light bulb that has a color temperature of approximately 3200 K. To maximize accuracy, this device also consists of variable optical filters that select the wavelength at which the solutions absorb the most light. The light that falls onto the photocell generates an electrical current directly proportional to the intensity of the light falling on it. This small electrical signal is increased in strength by the amplifier. Then, the amplified signal is passed to a galvanometer or digital readout, which is calibrated with a logarithmic scale, and the value can be read directly. The readings from the measured sample are compared with the reading from a pure sample solution that is used as a reference. The substances and chemical contents in the sample will absorb light for a variety of reasons, and they absorb the optical signal at a different wavelength. Thus, not only can the color of the sample be measured, but different chemical compositions can also be identified based on light absorption.

i. Nut Oil

Researchers have studied the methods for quality determination on various types of nut oil. One of the parameters that is measured for the quality determination of nut oil is color determination. Monounsaturated (MUFA) and polyunsaturated fatty acids (PUFA), as well as minor lipid components that affect the color of nut oil, play an essential role in human nutrition and health. Colorimetry is one of the popular methods for color determination of nut oils.

After each step of the refining process, the physicochemical properties of the oil could change, including its color [62]. The measurement of color after each step of the refining process is performed by using a chromameter or, technically, a photoelectric colorimeter, where the color is expressed as a CIE L*a*b* value. The procedure for color measurement in this study was performed by placing oil in a 1-cm cuvette, and a chromametric calibration plate was used as the background. The changes in color values of the oil can be observed in Table 9.

Santos et al. (2013) developed a low-cost colorimeter system [63] based on a report by Motta et al. (2005) where a wooden black-colored box consisting of a source of illumination formed by 21 high-brightness LEDs and a photodiode sensor was used. The colorimetric analysis and color analysis of Brazil nut oil obtained from different types of extraction was conducted using the developed colorimeter, where the light detected by the photodiode was expressed in CIE L*a*b* values. The results from the colorimetric analysis were used to validate those of the visual evaluations.

A comparison of the physicochemical properties, such as fatty acid compositions along with the color of the tiger nut tuber oil with olive, maize, sunflower and soybean oils, was investigated by El-Naggar et al. (2016) [64]. In this study, color was measured using the Lovibond method with a 13-cm cell. Concerning the color of the tiger nut, olive, maize, sunflower and soybean oils, the color parameters of 0.9, 3.1, 0.8, 0.8 and 4 were observed, respectively, in the red scale, while the yellow scale was fixed at 35. Table 10 shows the color differences between the studied oils obtained with the Lovibond method. The tiger nut tuber oil thus had a golden color that made it suitable for numerous uses in the food industry.

ii. Chia Seed Oil

For Australian chia seed oil, measurement of its color was conducted by using a photoelectric colorimeter with a white calibrating standard (*L* = 97.64, *a* = 0.16, *b* = 1.62) as a reference. The light detected by the chromameter is expressed in CIE L*a*b* values [65]. From the measurement conducted, the a* value of the Australian chia seed oil appeared to be considerably lower, while the b* value was higher than that of most common vegetable oils. The higher b* value of the Australian chia seed oil indicated a more intense yellow color and hence the presence of increased amounts of carotenoids. Three distinct maximum absorptions were observed at 425 nm, 450 nm and 475 nm, of which the peak at 450 nm was the strongest. The absorption peak indicates that the oil contained a higher quantity of carotenoids with traces of xanthophyll and chlorophyll.

#### 4.2.3. Optical Spectroscopy

The technique of optical spectroscopy has gained growing interest. It is becoming a successful analytical method for scientific study in many fields, such as food industries, automotive industries and agriculture industries. The optical spectroscopy studies focus on the phenomena of transmittance, absorbance, fluorescence or dispersion to determine the optical characteristics of matter [66]. In theory, each substance will have unique spectral properties that can be discerned from all the others. Some of the optical spectroscopy methods of detection are UV-Visible spectroscopy and fluorescence spectroscopy.

Optical spectroscopy is a method used to determine the optical characteristics of material interaction with the electromagnetic (EM) waves. The EM wave range includes gamma rays, x-rays, ultraviolet rays, visible light, infrared, microwaves and radio waves. When an EM wave interacts with matter, the matter absorbs, reflects, refracts, diffuses or emits various wavelengths of EM radiation or some combination of wave behavior.

##### UV-Vis Spectroscopy

**Figure 6:**
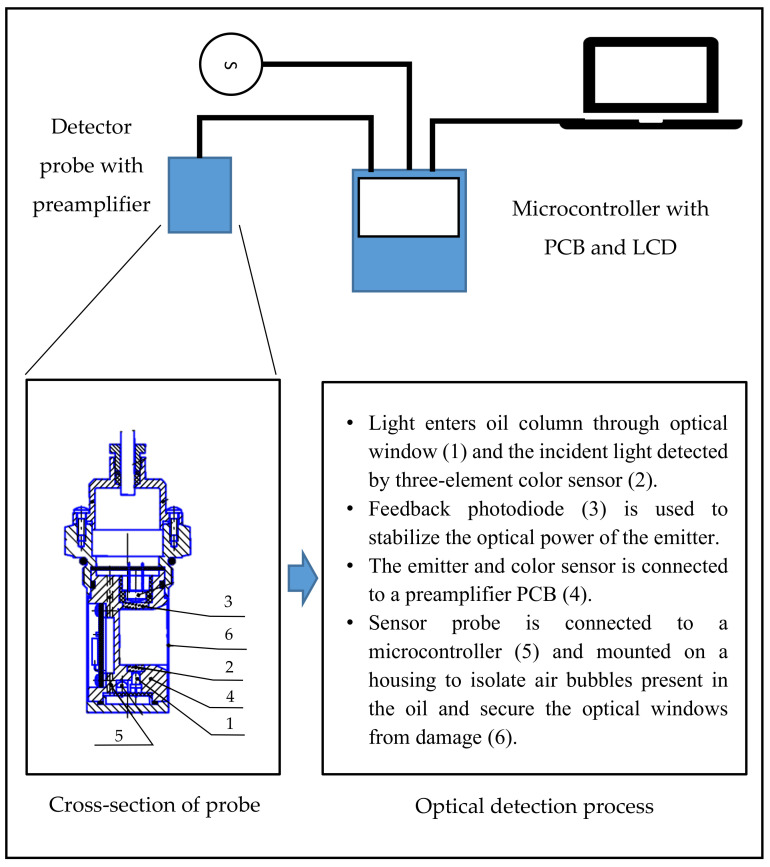
Instrumentation for CR detector application.

**Figure 7:**
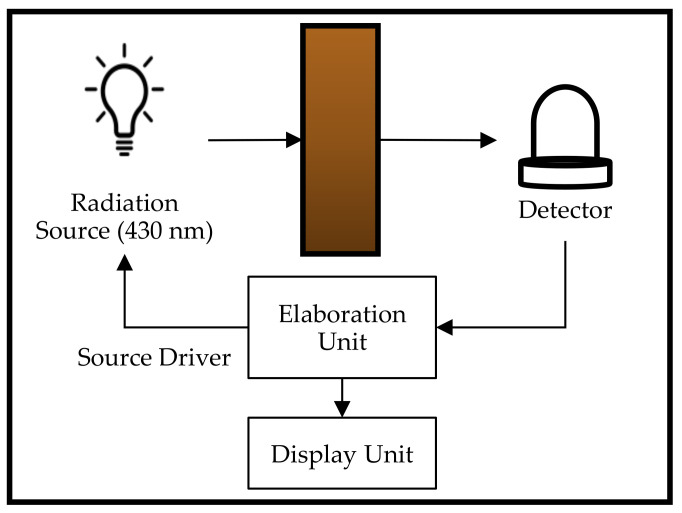
Block diagram of the proposed spectrophotometer.

(A) Mineral Oil
  - Diesel Oil
    The color of diesel oil was determined through the spectroscopy method in the range of 400–700 nm, where the relationship between the transmittance area and the color of the diesel oil in accordance with ASTM D 1500 was identified [67]. A model was used from the transmittance area values to create an analytical curve. Using this method for 505 diesel oil samples, 96.8% accuracy was obtained when the results were compared with the conventional method of a manual colorimeter.
    In another study, the color index of metropolitan diesel and interior diesel was determined through combined calibration with PLS to molecular spectrophotometry. In addition, the subjective characteristic of ASTM D 1500 can be eliminated through the UV-Vis spectroscopy method, even if there is a presence of red dye in the interior diesel oil [68]. Corgozinho et al. (2009) developed two calibration models of partial least square regression to correlate the predicted values and the real values of ASTM color [68]. It was found that the coefficients of linear correlation were 0.9956 and 0.9966 for metropolitan diesel and interior diesel, respectively. The findings obtained from both studies showed that there were significant differences in the transmittance spectra for a different color index of diesel oil. As the color index of diesel oil increases, the transmittance spectra decrease.
  - Transformer Oil
    An improvement on the UV-Vis spectroscopy approach related to ASTM D 6802 involves introducing an ANN to estimate the remnant lifespan of transformers [69]. The spectral response of the insulating oil can be instantly measured with cheaper equipment, and anyone in the same field can conduct the test. Leong et al. (2018) have proven the possibility of applying this technique to develop a portable device that can measure the color index of transformer insulating oil on-site in accordance with ASTM D 1500 [11,70]. The regression technique was used to obtain the best representation of the relationship between the absorbance, cutoff wavelength and color index. A mathematical model based on the regression analysis was developed that described the relationship between the area and color index of the transformer oil as in Equation (7):
    CI = (9.017 × 10^−3^) Area (7)
    It was found that the prototype produced accurate results in determining the color index of the transformer insulating oil with a smaller error in the color index compared with the conventional method of using a visual color comparator, with an RMSE of 0.6274.
  - Lubricant Oil
    Refined oil using active Ca-bentonite was evaluated using the spectroscopy technique by scanning in the wavelengths of 200–1200 nm [71]. The effect of the active substances can be observed in the refined oil’s color. However, the focus of this study was to identify the content of the refined oil.
    In another study, two single-wavelength LEDs at 590 nm and 840 nm were used to measure the color of lubricant oil according to the ASTM D 1500 color scale [72]. Two sets of optical systems were designed using two sets of light-dependent resistors (LDRs) and LEDs. One set was used to measure the color of the lubricant oil, while another set was for measuring the turbidity and to correct the deviation in color scale readings due to turbidity. As the color index of the lubricant oil increases, the light intensity at the LDR decreases. The embedded system reads the transistor–transistor logic pulses from the sensors, counts the pulse frequency and converts the number into a color scale before displaying the results on the LCD. The different color indexes of lubricant oil can be differentiated, as different colors will give different frequencies. The schematic diagram of the experimental set-up can be found in [72]. This method eliminates the use of a spectrometer, making the system compact.
    Another system to monitor the color of engine oil only makes use of a single light source, turbidity sensors and microcontrollers to measure the amount of light absorbed by the oil [73]. The light source used was a white LED placed directly facing the sensor with the oil samples in between. The sensor measured the amount of light that was scattered by particles in the oil. The turbidity level of the engine oil increased as the amount of particles in the oil increased. This was due to more light being scattered by the particles in the oil. Then, the microcontroller processed the value from the sensors and lit up an LED to indicate the condition of the engine oil. However, no accuracy testing or further analysis was conducted in this study to support the invention.
  - Hydraulic Oil
    A color change detecting device for synthetic hydraulic (SHD) oil was developed using LEDs as the light source and photodiodes of three sensing elements [74]. The color ratio (CR) and total contamination parameters based on the transmitted light intensity passing through the oil were used for chemical contamination assessment. The color sensors detected the light and measured the luminous intensity in red, green and blue wavelengths.
    Figure 6 shows the diagram of the instrumental set-up for the CR detector application. However, for the SHD-1 oil, the CR showed a more unambiguous indication of oil degradation compared with the total contamination index (TCI) detected by all three RGB wavelengths shown in [74]. This shows that color is vital in determining the condition of the oil. TCI-R is the total contamination index for red-wavelength, TCI-G is the total contamination index for the green wavelength, and TCI-B is the total contamination index for the blue wavelength.
(B) Vegetable Oil
  - Olive Oil
    Olive oils produced from different origins have different color spectra [75], where color is statically analyzed and correlated to the phenolic compounds and environmental parameters. Color measurement using the spectroscopy method, where the color is expressed in CIE L*a*b* values, is commonly used [15,76,77,78]. A 5-mm path length cell was proposed, as it allowed reliable measurements of even the darkest color of virgin olive oil samples [77]. A different thickness of the oil sample holder can also affect the light measured [15,77]. Furthermore, the study on the color of olive oil through characteristic vector analysis was applicable to the reconstruction of olive oil sample transmittance spectra, and the use of the transmittance spectra was sufficient for a close approximation when the measurements were made with 5-mm path length cells [15].
    A method for the numerical description of virgin olive oil with only two absorbance measurements at 480 nm and 670 nm from a spectrophotometer was also proposed [76]. The light detected was expressed as CIE L*a*b* values, and the polynomial expressions were obtained with chromatic coordinates and chroma as input data. olive oil.
    Table 11 shows the equations developed for estimating the chromatic coordinates and the chroma *C* of the olive oil.
    A new color scale standard, the Modified Uniform Oil Color Scale (MUOCS), was developed based on a unique set of 1700 virgin oil samples [79]. This newly proposed standard has 60 color scales, which consist of a 3-number code representing lightness (1–14), green (2–16) and yellow (1–14) values. This MUOCS is an improved version of the previous color scale, the Uniform Oil Color Scale (UOCS), and Bromthymol Blue (BTB). A portable device that adopted this new color scale was developed as shown in [79]. This device used a white LED as a light source and a detector sensitive in RGB regions that simultaneously measured the RGB color coordinates of the incident light. Although this device can measure oil in the MUOCS accurately, the differences are significant when compared with the results obtained from the commercial spectrophotometer.
  - Palm Oil
    The color of palm oil not only affects the customer preferences [80] but also serves as an indicator of the levels of the chemical contents, such as the free fatty acid content [24,53,80,81] and total carotenoids [24,82]. These chemical contents determine the quality of the palm oil after a certain refining process. The overall minimum requirement for the free fatty acid content (as palmitic acid) in crude palm oil (CPO) set by the Palm Oil Refiners Association of Malaysia is 5%. They should be less than 0.1% in RBD oil [83].
    The automated colorimeter based on the UV-Vis spectroscopy technique was proposed by Tan et al. (2004) to determine the Lovibond color of palm oil, refined palm oil (RPO) and refined palm olein (RPOo) [80]. Three light sources of red (R), green (G) and blue (B) LEDs were used with a microprocessor to process all the digital signals and correlate with the Lovibond color units (red and yellow units). The values obtained from the signal processing were compared with the predetermined scale of the color and path length. The readings from the proposed colorimeter were calibrated, and appropriate software using an ANN was used to calculate the Lovibond value. The comparison between the automatic and manual instrument measurements showed an acceptable R^2^ value of 0.9236 for the red color and 0.7974 for yellow. The developed automated colorimeter instrument showed good results in determining the color of palm oil according to the Lovibond scale. The color analysis was carried out according to ASTM D 6045 using a UV-Vis spectrophotometer. The CIE L*a*b* values were analyzed to study the effect of bentonite on the flashpoint, density and color as refining agents in waste cooking oil [84]. However, a clear relationship between the changes measured in the flashpoint, color and the composition of the waste cooking oil after bentonite was added could not be easily identified.
  - Sunflower Oil
    An in-line measurement system of sunflower oil color using 8 LEDs was developed [85]. This allowed measurement of transmission and reflection at four wavelengths: red (R), green (G), blue (B) and near-infrared (NIR). The RGB light was used to obtain the sunflower oil color, while the NIR light was used to determine the bubble-related scattering effect. A block diagram on how the in-line system works can be found in [85]. The direct method and indirect method were performed for the calibration of the in-line equipment with a Lovibond tintometer.
    The system produced similar results to the ones obtained by trained operators through a visual colorimeter and automatic colorimeter [85]. The acceptable corresponding values from the analysis of data obtained through the proposed system and visual and automatic colorimeters are shown in Table 12.
  - Nut Oil
    The impact of roasting on the composition and quality of cashew nut oil was investigated by Liotrakoon et al. (2016) [86]. The total color change value was calculated based on the standard definition of the CIE L*a*b* color difference to observe the differences in the oil color with and without the roasting process. The oil that was extracted from the roasted nut was browner and affected the oil content. The equation for this determination is shown in Equation (8):
    (8) $$\Delta E_{ab}{}^{*}=\sqrt{{\left(L_{0}^{*}-L^{*}\right)}^{2}+{\left(a_{0}^{*}-a^{*}\right)}^{2}+{\left(b_{0}^{*}-b^{*}\right)}^{2}}\;$$
    where $L_{0}^{*}$, $a_{0}^{*}$ and $b_{0}^{*}$ denote the color parameters of the unroasted oil and L*, a* and b* indicate the color parameters of the roasted oil. It was found that the total color change value increased from 3.92 to 8.18, showing that the roasted cashew nut oil was darker than the unroasted cashew nut oil. The higher the total color change value, the bigger the difference in color.
    However, the roasting process of the nut caused significant losses to the bioactive compound in the oil. Al-Bachir (2015) investigated the effect of gamma radiation and storage on the peanut oil characteristics and found that the color was only affected by the storage period [21]. A spectrophotometer colorimeter with CIE L*a*b* values was used for color determination in both studies.
    A comparison of two methods for measuring the quality of walnut oil, where one of the parameters for quality assessment was the color of the oil, was conducted [87]. Table 13 shows the standard color for walnut oil. The Lovibond colorimeter and spectrophotometric method with absorbance measured at several wavelengths were investigated. The photometric color index (PCI) was calculated using Equation (9) with the data obtained from the spectrophotometric method:
    PCI = 1.29 (A_460_) + 69.7 (A_550_) + 41.2 (A_620_) − 56.4 (A_670_) (9)
    where PCI is the photometric color index and A is the absorbance reading at wavelengths of 460 nm, 550 nm, 620 nm and 670 nm. Physicochemical and biochemical changes could be quickly determined using the Lovibond and photometric methods.
  - Linseed Oil
    The color of linseed oil was evaluated to observe changes in color during periodical heating [19]. Measurements using a spectrophotometer in the visible wavelength range (320–700 nm) was performed. The area under the absorption curve indicates the concentration of the coloring matter in the oil. Sixteen readings of optical density were obtained from 400 nm to 550 nm, with a resolution step of 10 nm. The sum of the 16 readings was then multiplied by 10 to give the approximation for the area under the absorption curve. Two groups of linseed oil samples (stripped and non-stripped) were analyzed individually in triplicate. The results were reported as the mean ± SD. Table 14 shows the color indexes of both groups of samples of linseed oil as measured, where the values are presented as the mean ± SD. The color index was based on a previous study [88] and not a color scale introduced by any authorized bodies.
(C) Honey
  The color of honey ranges from colorless to amber tones and almost black. Usually, bright yellow, greenish or reddish colors are more typical for honey. The color of honey depends on the nectar that has been gathered by the bees. This is because different nectar has different organic and chemical compounds. The color of honey, however, can change with time when exposed to high temperatures [35]. Honey becomes darker when stored at a higher temperature. After some time, the stored honey may granulate, producing crystals. The different sizes of crystals produced can affect the color of the honey. Many variables causes the variation of honey’s color, such as its origin and bee species, in addition to being affected by the weather, processing, packaging and storage [89].
  Al-Farsi et al. (2018) measured the absorbance at a wavelength of 635 nm [90], while Moniruzzaman et al. (2014) measured the absorbance at 450 nm and 720 nm [91] to determine the color intensity by using a spectrophotometer in accordance with the Pfund scale. A positive correlation between the color, flavonoids and phenolics was observed in both works. The color intensity of the honey increased with higher phenolic and flavonoid contents in the honey. Table 15 shows the correlation established by Al-Farsi et al. (2016) between the color, flavonoids, phenolics and antioxidants. It was found that the darker color of honey was due to the presence of high concentrations of flavonoids and phenolics that increased the number of antioxidants, thus indicating the better qualities of the honey.
  Another study also measured the color of honey, expressed in CIE L*a*b* values. The results were compared with the conventional method using Lovibond with different background colors [92]. The CIE L*a*b* values obtained from the light intensity based on the UV-Vis spectroscopy technique managed to obtain a strong correlation between the phenolic content and antioxidant activity of honey [93]. Other than that, it was also useful for identifying different types of honey. The honey samples were divided into two groups—bright honey (black locust, goldenrod, rapeseed and lime) and dark honey (buckwheat and heather)—as shown in Table 16. Based on the analysis in correlating the CIE L*a*b* values with the phenolic content and antioxidant activity, good R^2^ values of 0.9383, 0.9235 and 0.9116 for the L*a*b* values were obtained.
(D) Maple Syrup
  Different grades of maple syrup have different usages in the food industry. Each color grade of maple syrup has unique flavor characteristics. Therefore, the color determination and color grading of maple syrup plays an important role in the customer’s decision-making process.
  Measuring color by calculating the light transmittance at 560 nm using a spectrophotometer has been studied by several researchers. This was to investigate the effect of air injection on the color [20], investigate the effect of processing parameters (residual pressure or vacuum in the evaporator-crystallizer, mixing speed of the massecuite and crystal growing time) on the crystallization yield, sugar particle size and color of the final sugar [94] and investigate the effects of processing, the production site and the harvesting period on the quality variation and its relationship to the microbial population [95]. Although these studies were meant to investigate the different effects of processing parameters on the maple syrup color and condition, the color measurement method followed the standard protocol introduced by the USDA [37]. The percent of light transmitted through the sample was measured with a spectrophotometer using a 10-mm cuvette at a wavelength of 560 nm.
(E) Beer
  Finding tristimulus and chromaticity values using light transmission measurements at several wavelengths were first proposed in 1992 by S.M. Smedley [96]. Transmission measurements were conducted at 360 nm, 450 nm, 540 nm, 670 nm and 760 nm on an undiluted beer in a 25-mm path length cell. Curvilinear interpolation was applied to the measurements to generate a set of calculated transmission values at 10-nm intervals in the range of 360–780 nm. Lastly, the tristimulus values were calculated from the set of interpolated transmission values. By using the CIE color matching functions for a secondary observer and the spectral power distribution for CIE Standard Illuminant C., the tristimulus values could then be converted into the desired subset parameters, such as CIE L*a*b* values.
  Since the utilization of tristimulus values to measure the color of beer has been introduced, many studies proceeded to use the same CIE L*a*b* values. However, there are still differences in terms of measurement methods. One study on the characterization of beer using diffuse light absorption spectroscopy in visible and near-infrared bands. The light detected was expressed in tristimulus color values in the form of a data matrix, and correlation with turbidity and a refractive index was created [97]. The classification of the beer was determined according to its class. The schematic diagram of the set-up for diffuse light absorption spectroscopy using optical fiber technology can be found in [97]. Nearly all the light reflecting on the surface of the sphere is diffusely reflected, and the addition of an absorbing sample in the cavity results in a decrease in the sphere’s radiance, regardless of other scattering effects caused by suspended particles in the sample.
  Although color determination through the absorbance measurement at 430 nm shows promising results, the other wavelengths that were not detected by the spectrophotometer seem impractical. An investigation on utilizing a single-wavelength LED with a peak wavelength of 430 nm as the light source for a low-cost spectrophotometer for the measurement of beer color based on the EBC method was performed [98]. The device used a photodiode as the detector with a peak spectral response at 436 nm, and a microcontroller was used to obtain the calibration curve equation. A power regression was chosen as the calibration curve, obtaining a coefficient of determination R^2^ of 0.9677 for the V_LOW_ forward voltage and 0.9963 for the V_HIGH_ forward voltage. V_LOW_ and V_HIGH_ are the luminous intensity for the radiant source. Corresponding to a forward voltage of the LED, V_LOW_ = 3.3 V for the lower intensity, and V_HIGH_ = 4.0 V for the higher intensity. Figure 7 shows the block diagram of the proposed low-cost spectrophotometer.
  Koren et al. (2020) found that the color determination using CIE L*a*b* color space parameters calculated from the transmission spectra measured in the visible range (380–770 nm) could identify beer color more objectively compared with the absorbance method [99]. This was due to a clear difference between the transmission spectra for each beer that could be observed through the CIE L*a*b* values and the different raw materials in the beers, which contained various coloring compounds. It was found that one wavelength measurement from the absorbance method was insufficient to describe the accurate color of a sample, even if it was a traditional beer type. The color of the beer was first determined according to the standard Analytica-EBC color-measuring method. The transmission spectra were obtained using a spectrophotometer. The tristimulus values and chromaticity coordinates of the samples were calculated from the transmission spectra as defined in the CIE 1931 standard colorimetric system based on the description of the Commission Internationale de l’EćlaiZrage. The values were then converted into CIE L*a*b* values. With the CIE L*a*b* values, the absolute L*a*b* color between two samples was calculated and then compared with the EBC values. Table 17 compares the EBC difference and absolute L*a*b* differences of the beers. From the table, the absolute L*a*b* difference between the same beer category showed a larger value compared with the EBC difference. Zhu et al. (2013) found that if the absolute L*a*b* values were less than 1.5, it meant that there was almost no difference, and if the absolute L*a*b* values were more than 1.5, it meant that there was a slight difference. Meanwhile, if the absolute L*a*b* values were more than 3.0, it meant that there was some difference, and if the absolute L*a*b* values were larger than 6.0, it meant that there were significant differences between the colors of the beer samples, although they were in the same category [100].

##### Fluorescence Spectroscopy

Fluorescence is when an object or material absorbs photons at a particular wavelength or energy, and after a few nanoseconds of excitation, it emits photons at a longer wavelength or lower energy. Fluorimetry or fluorescence spectroscopy uses a light source in the range of 180–800 nm. The light is passed through a sample in a cuvette. The output, where the sample emits the light, is measured at an angle. In this method, the light that is absorbed by the sample and the light emitted by the sample can be measured [101,102]. The concentration of the chemical composition is directly proportional to the intensity of the light emitted. 

**Figure 8:**
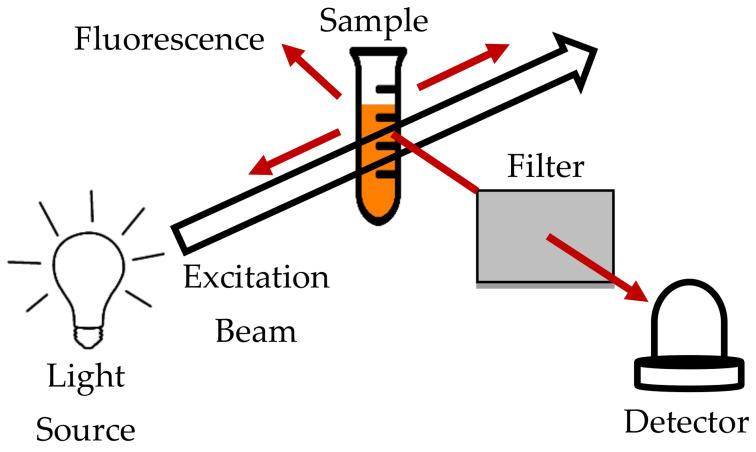
Fluorescence schematic.

Figure 8 shows the schematic of the fluorescence method.

However, the chemical compound that has a photochemical reaction at the wavelength range of the fluorescence spectroscopy method is not suitable to be examined. This selectivity of the conventional fluorescence method appears to be inadequate. A multidimensional method, such as synchronous fluorescence (SFS) and excitation emission matrix fluorescence (EEMF), is to be used in liquid quality determination, as it can provide more information about the sample’s chemical composition [103].

Color can be accurately predicted by applying specific mathematical model analysis such as PLS regression using the fluorescence spectra [101]. Through this method, different emission spectra peaks can be observed, as different peaks will carry different information regarding the measured sample. Since the fluorescence method was conventionally used for chemical compound identification, a correlation between the chemical compound and color can also be established.

**Figure 9:**
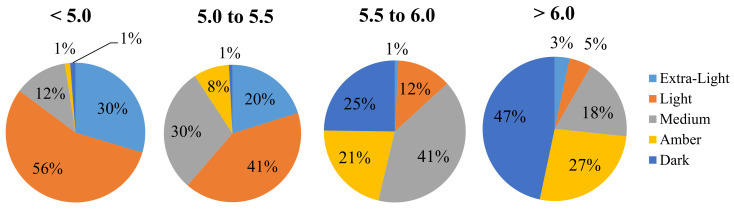
Distribution of syrups according to color grade in different classes.

The dark color of maple syrup is a defect caused by microbial contamination. The intrinsic fluorescence method for the measurement of the physiochemical properties of maple syrup produced a good accuracy of R^2^ = 0.91 and R^2^ = 0.88 while providing valuable semi-quantitative information on the pH and microbial counts of sap [101]. The proposed method showed good potential for practical application, since it could also be used to identify the chemical component in the sample. Prediction of the maple syrup grade (color) and sensory quality through estimating the microbial quality using the adenosine triphosphate (ATP) bioluminescence method was also performed [104]. In this study, it was found that the color and flavor could be assessed using the proposed method because of its relationship with the contamination level. Figure 9 shows the distribution of syrups by color grade in different classes of sap contamination obtained by bioluminescence. Lighter-grade syrups were produced mainly from low-contamination saps, while darker-grade syrups corresponded predominantly to high-contamination saps.

##### Image Analysis

Image analysis makes use of images taken from a camera in the set-up and extracts the color information from the image. For example, the color information of the image can be in the form of CIE values. The CIE values involve various color spaces, and each of the color spaces is formed by a linear or non-linear transformation of the RGB color space [105] or CYMK color space. This method can produce a fast and objective measurement of the liquid.

Researchers use the color information obtained from the image analysis as the input for artificial intelligence (AI) models for quality classification, identification or prediction of chemical contents. Many types of AI have been used depending on the objectives of the study, such as ANNs [53,81,106] and Support Vector Machines (SVMs) [107,108]. With the use of AI, the accuracy for color determination can be increased due to sufficient training data and the ability of the AI to self-learn and improve without being explicitly reprogrammed by the developer if new information is obtained.

**Figure 10:**
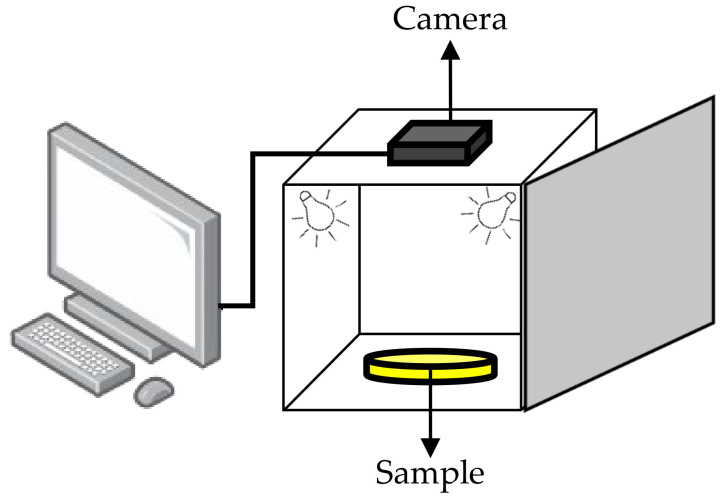
Set-up for the image analysis method.

The set-up for conducting image analysis is shown in Figure 10. A few components of the system, such as the light source and the digital camera, need to be inside a confined box to prevent external light from entering. The interior of the walls of the box was covered with white paper to avoid light scattering. The camera was connected to the computer as shown in Figure 10, and image analysis software was used for color identification. For better results, a high-resolution camera was recommended so that a clear image could be obtained for analysis. Only the center region of the picture taken was selected for analysis [106]. Typically, the information in the form of CIE values would be correlated with the chemical attributes of the liquid. The correlation between the reference sample color and the testing sample color was first analyzed to verify that the colors were the same. Then, a relationship between the color information obtained and the chemical content could be established. From this, the quality and condition of the liquid could be determined.

**Figure 11:**
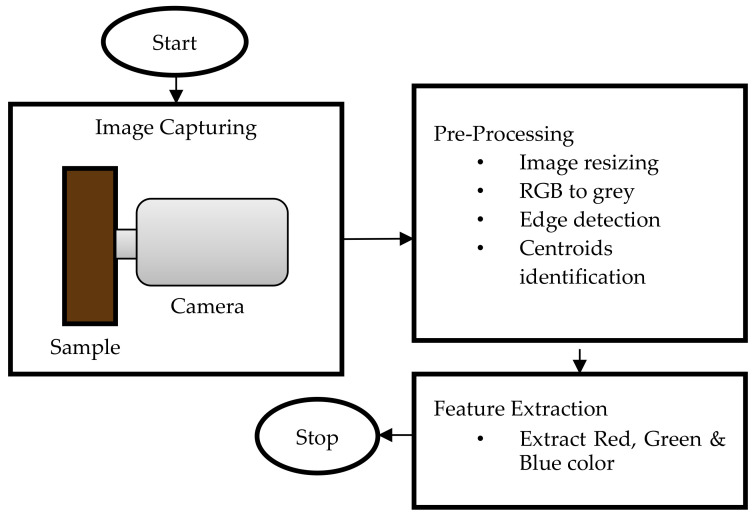
Set-up for palm oil image processing [81].

**Figure 12:**
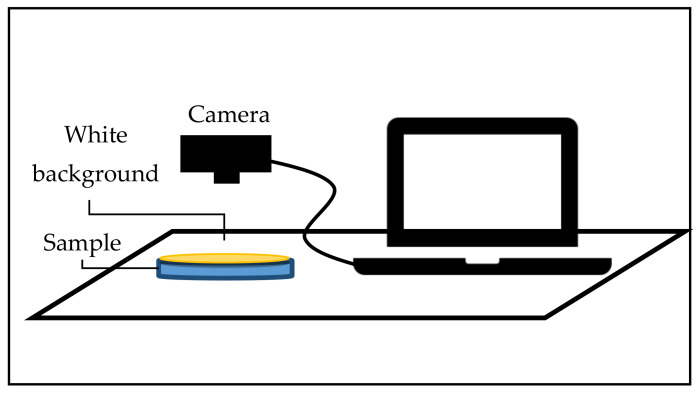
System to capture images of oil.

**Figure 13:**
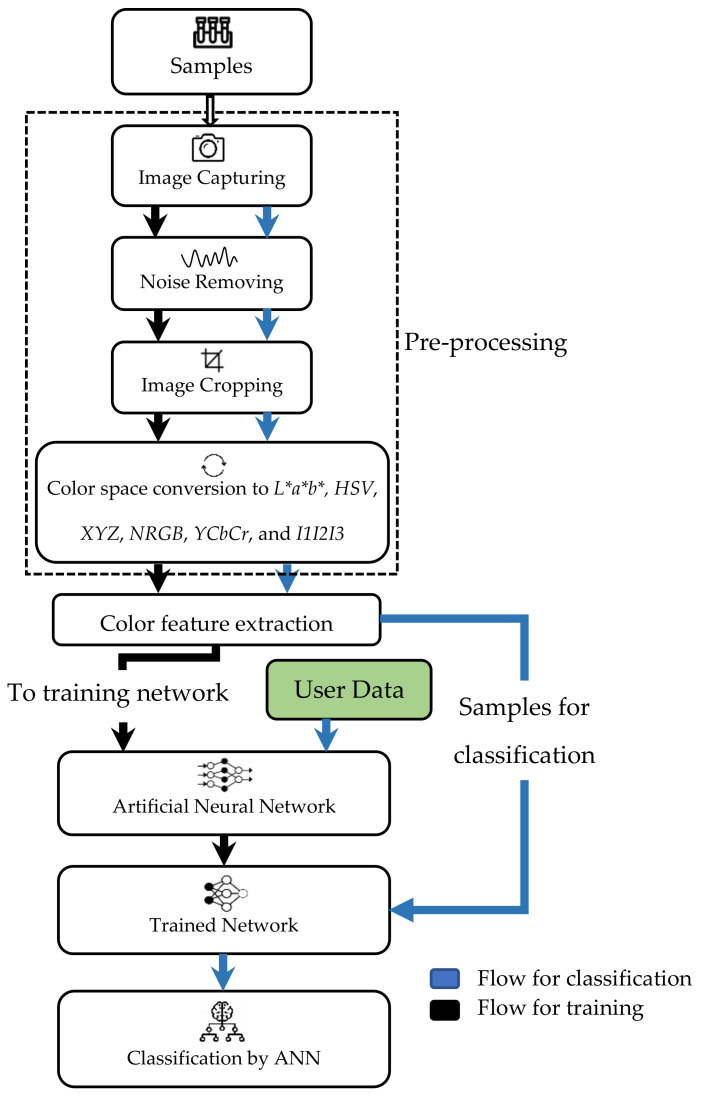
The main steps in the development of the program for honey classification using machine vision.

**Figure 14:**
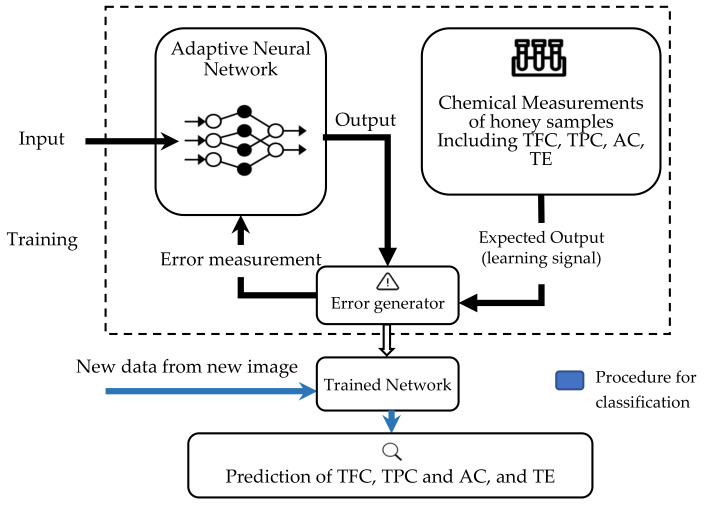
Schematic description of the method for prediction of honey characteristics.

**Figure 15:**
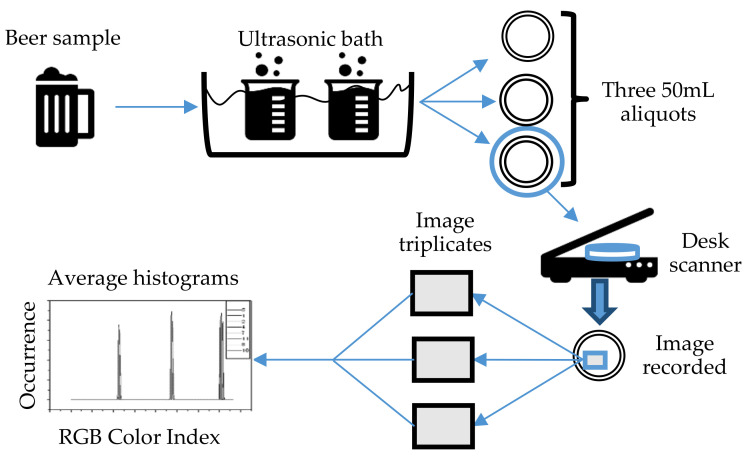
Procedure for obtaining the image data matrix.

Other than that, the color information in the CIE L*a*b* values was also used to relate with the color standards such as Pfund, Lovibond and ASTM. The correlations between the color information obtained and the color standard can be observed after further mathematical analysis is performed.

(A) Lubricant Oil
  A study by Anacan et al. (2018) investigated developing a machine vision system that scanned a car oil’s engine using LabVIEW software through image processing [109]. The system computes the light intensity from three LEDs (white, red and blue) reflected from the measured sample. The color frequency intensity (CFI) sensor was designed to measure the light intensity of the LEDs. The system developed by Visual Instruments in LabVIEW will then automatically show the status of the engine oil in terms of color, light intensity that can pass through it and its viscosity. The diagram of the system set-up for image acquisition of the engine oil samples can be found in [109].
(B) Vegetable Oil
  (i) Palm Oil
    Image analysis enables the color detection of cooking oil, where it reads the RGB color values of the sample [81]. Images were taken by an RGB camera and processed in MATLAB software for the extraction of RGB values as shown in Figure 11. The images were then preprocessed by resizing the sample images to obtain uniformity, followed by converting the colored image to grayscale before the edge detection operation. Edge detection is a process for finding the boundaries of objects within images. The preprocessing stage is to ensure the pictures obtained have the same dimension and traceable boundaries before extraction of the sample’s RGB color. In this study, two simple feed-forward ANN models were used to classify the quality of reused cooking oil. One model used TPM, organic vapor gas and methane as inputs, while the other model used the three RGB values as inputs.
  (ii) Cotton Oil
    Cotton oil is commonly used as a high-quality standard for measuring the quality parameters of other vegetable oils, such as flavor and odor quality. Its mild taste and odor give features of virtually every imaginable food application. The degree of cotton oil’s color depends on the stage of the refinement process like most oils. The six phases of the cotton seed semi-refined process, along with its changing color, are shown in Table 18.
    The primary colors of red, yellow, blue and neutral were analyzed for the classification of cotton oil using image processing techniques through the oil color [110]. The colors were extracted from the image captured using the system based on Figure 12. From the color information obtained, the classification of the oil was performed using several classifier models such as K-Nearest Neighbor (KNN), Quadratic Discriminant Analysis (QDA) and the Extreme Learning Machine (ELM) [110]. An ELM neural network produces the highest average accuracy of 95.8% due to its capability to learn based on the training data provided.
    Table 19 shows the percentage of accuracy for each model in the classification of the cotton oil based on the stages of the semi-refining process.
(C) Honey
  The image analysis technique to determine the color of honey has also been studied by Shafiee et al. (2013) [106] and Dominguez and Centurión (2015) [36]. Shafiee et al. (2013) used machine vision to assess the color of honey that is independent of the individual tester’s experience [106]. The machine vision system consists of a light source, an image capturing device, an image capture board and appropriate computer hardware and software. The image processing part involves image acquisition and smoothing and the conversion of RGB to a different color transformation. The post-processing part includes applying an ANN to achieve classification and prediction of several chemical components. Figure 13 and Figure 14 show the steps for the development program for honey classification using a machine vision system and a schematic diagram of the method for prediction of honey’s characteristics, respectively.
  However, Dominguez and Centurión (2015) proposed an analytical method based on digital image analysis combined with multivariate calibration to obtain a fast and objective measurement of honey’s color [36]. The Pfund color grader was used as the color reference for developing this technique. An image capturing device was developed where a camera was placed directly on top of the sample in the center of a circular daylight fluorescent lamp. Digital images from different samples of honey with different Pfund color values can be found in [36]. Through this technique, the illumination and the distance between the sample and the camera remained constant. The interior wall of the box was covered with white paper to prevent any light scattering.
  Data analysis of the images captured was conducted using selected regions of the images and histograms for each color channel. Table 20 shows the obtained results for the calibration models of honey samples in the test set using RGB, HSB and grayscale color models. The root mean square error of calibration (RMSEC) is the measurement of the average difference between the predicted and measured response values in the calibration stage. The root mean square error of cross-validation (RMSECV) is the measurement of the average difference between the predicted and measured response values at the validation stage, which explains the ability of the model to predict the color of a new sample. The bias also can be defined as the mean of the errors. Based on the table, the best calibration model was achieved by using the HSB model. Good correlation can be observed between the values obtained with the reference method and those calculated by the digital images of the HSB color model with an R^2^ value of 0.97 and root mean square error of prediction (RMSEP) of 2.46 [36].
(D) Beer
  Other than the optical spectroscopy method, image analysis for color identification has also been investigated [6,111,112]. The information obtained from the images was used as the data for pattern recognition to categorize the beer into its color index according to the RGB channel [112]. Figure 15 shows the procedure for obtaining the image data matrix of the beer. Histograms of the frequency distribution of color indexes according to each RGB color channel were obtained for each digital image and decomposed into vector lines, with each vector having 256 components (indexes or color tones). Digital images of beer can be used to classify different brands of beer of the same type or category. The average histograms of the R, G and B color channels produced similarity patterns in the principal component score plot obtained from images produced on a desk scanner. The magnitude of the color index accompanied the color shifts in the principal component graphs in the principal component axes as a consequence of the average tone for each brand.
  Another color determination method has been investigated where the colors of beers were determined in two color scales—*XYZ* and CIE L*a*b*—through a spectrophotometer and digital imaging [111]. The beer color was first modeled by type (light, dark, fresh or with lemon) through step-by-step linear discriminatory analysis. K-means clustering was used to compare and classify the tested types of beer based on their commonality. Correlation and discriminant analyses were conducted to establish the types of beer, including the unknown samples. The proposed model can identify the types of beer and determine the closest group of beer that is not used in the model.

#### 4.2.4. Issues and Challenges

Table 21 presents the summary of the analysis on the methods and techniques for color determination of liquids reported by previous studies. The color determination methods of different amber-colored liquids were tabulated with their techniques, detection wavelengths, accuracy and other factors. From the summary, it is clearly shown that objective methods have higher accuracy compared with subjective methods. However, the issue is that the accredited laboratories continue to utilize the conventional methods, which are mainly subjective methods, because the authorized bodies that introduce the standard are not adopting most of the methods proposed or reported by the researchers. Aside from the inertia to maintain the status quo, the other major factors which lead to the slow adoption of new technologies include the high initial investment for laboratory equipment, the complexity of measurements and the lack of data validated mutually by the technology inventors and users.

In addition, these newly reported methods were derived based on high-end equipment that is neither portable nor low in cost. Trained personnel are needed to conduct the measurements, since most of the methods involve complex processes and procedures. Therefore, the samples need to be transported to the laboratory from the site.

Most of the research in objective methods focuses on the chemical compositions of the liquids, such as chlorophyll, phenolic content, dissolved gas, total pigment content, water content and oxidation by-products, which are then related to the color of the sample. Although this method is accurate because no human judgment is involved, many other experiments are also required to identify the condition or quality of the liquids. The methods or studies do not focus on color determination, and color was only measured as an additional parameter to be reported in a color scale, thus increasing the cost of the color measurement of the oil.

## 5. Recommendations and Future Prospects

To overcome these issues and challenges, the ultimate aim is to have a technique that can reduce or eliminate human intervention for quantification of samples. This is to minimize human error that can cause inconsistencies and inaccuracies in the measurements. The technique should also measure the color without any complicated procedure so that any operator can conduct the measurement. The ease of measurement leads to adoption of the new technique to replace or complement the conventional measurement methods or standards. It has been shown that UV-Vis spectroscopy is one of the popular techniques studied by researchers, as there are many research works investigating the capability of this technique in determining the color of a liquid. Measurement using the UV-Vis spectroscopy technique can be advantageous, as it can convert the measured spectrum of absorbance or transmittance into any preferred color scale.

In addition to utilizing the full spectrum from UV to the visible range, there is work reporting on the utilization of single-wavelength LEDs to determine the color of a liquid. This method has greatly reduced the cost of optical light sources. However, since an LED has a wide bandwidth compared with a laser diode, the accuracy of the measurements needs to be improved through a more sophisticated technique, such as frequency-sensitive signal processing. In this context, a single-wavelength laser diode or the hybridization of two or more narrow linewidth laser diodes can be proposed to measure the color of a sample.

Other than that, the system must be portable so that the measurement can be performed in situ to reduce the time and cost. Although there is real-time measurement or in-line measurement that has been investigated by researchers, it is still not widely used. Conventional methods are still the commonly accepted methods, possibly due to the accuracy and reliability of the alternative methods, which are not fully validated. The system and technique should have high accuracy and be in accordance with a certain color standard that has been introduced by the authorized bodies. Therefore, research work that is targeted at designing and developing a measurement device must be verified by comprehensive comparison and analysis based on the well-established color standards.

With the increasing computational power of microprocessors, the computation of data for prediction and classification can be carried out within milliseconds. To increase the accuracy of a newly developed technique, the implementation of machine learning and AI in the system to process and classify the obtained electrical signals is essential, as it can perform self-learning to increase the accuracy of color determination. Furthermore, by fully exploiting the computational power, the dependency on hardware can be greatly reduced, leading to a simpler hardware design with a reduced number of components. With less hardware or components, the cost for maintenance of the system can also be minimized.

## 6. Conclusions

The color of a liquid product is an important parameter that determines the product quality and marketability. An accurate technique for color measurement and determination is crucial to reduce the dependency on hardware utilization and human interpretations. In line with this effort, the discovery of a new technique may contribute to a better color grading system and standards. This review has carried out an extensive assessment of different methods for color measurement of amber-colored liquids. It is clearly demonstrated that although conventional methods can measure color and give satisfying results, automating the process through more advanced and objective methods that eliminate human involvement is the feasible way forward. Further advancement of color measurement techniques is required to obtain efficient future operation, integration and assessment. This review also presented the advantages and disadvantages of various types of color measurement methods in terms of equipment, materials, technique, accuracy and duration. It was found that the UV-Visible spectroscopy method has increasing popularity in this research field, as the data can be correlated with most of the established standards or color scales available. The wavelengths in the UV-Visible range can also be used to identify chemical components inside the liquid. Lastly, this review has identified several challenges and a few possible advancements for future research, which are listed below:

1. Development of functional devices based on research outcomes to increase the probability of adoption by industries;
2. Collaboration among researchers, accredited laboratories, standards or associations and industries in the validation and adoption of new technology;
3. Focus the research work on an optical spectroscopy technique that utilizes single-wavelength or dual-wavelength approaches instead of the full visible waveband so that the optoelectronic components and cost can be significantly reduced;
4. Fully exploit the computational power of modern microprocessors toward achieving the aim of minimizing the hardware and optoelectronic components.

## Figures and Tables

**Table 1:** Lovibond RYBN color range.

Color	Range
Red	0–79.9
Yellow	0–79.9
Blue	0–39.9
Neutral	0–39.0

**Table 2:** Pfund color scale [48].

Color Name	Pfund Scale (mm)
Water white	<9
Extra white	0–17
White	18–34
Extra light amber	35–50
Light amber	51–85
Amber	86–115
Dark amber	>114

**Table 3:** Beer color with units.

Beer Color													
SRM	2	3	4	6	8	10	13	17	20	24	29	35	40+
EBC	4	6	8	12	16	20	26	33	39	47	57	69	79

**Table 4:** Canadian federal classification of maple syrup [51].

Class	Grade	Description
Canada 1	Extra Light Light Medium Amber Dark	(a) Must not ferment; (b) Must have a clear and uniform color; (c) Its color grade must be extra light, light or medium; (d) Must have a maple flavor that is typical of its color grade and be free of any unpleasant odors or flavors.
Canada 2	Amber	(a) Must not ferment; (b) Must have a clear and uniform color; (c) Its color grade must be amber; (d) Must have a maple flavor that is typical of its color grade and be free of any unpleasant odors or flavors.
Canada 3	Dark	(a) Must not ferment; (b) Must have a clear and uniform color; (c) Its color grade must be extra light, light, medium, amber or dark; (d) Must have a maple flavor that is typical of its color grade and be free of any unpleasant odors or flavors.

**Table 5:** Quebec provincial classification of maple syrup.

Class	Grade	Description
1	AA (Extra Light) A (Light) B (Medium) C (Amber) D (Dark)	(a) Has a clear and uniform color; (b) Has a flavor that is typical of maple syrup; (c) Does not contain any flavor of caramel or sap or traces of insoluble calcium malate; (d) Shows only traces of crystallization.
2	AA (Extra Light) A (Light) B (Medium) C (Amber) D (Dark)	(a) Clear in color; (b) Has a flavor that is typical of maple syrup; (c) Shows only traces of crystallization; (d) Has any of these defects: flavor of caramel or sap or traces of insoluble calcium malate.

**Table 6:** “Grade A” color of maple syrup.

Grade A Color Classes	Flavor	Light Transmittance (%)
Golden	Delicate	≥75.0
Amber	Rich	50.0–74.9
Dark	Robust	25.0–49.9
Very Dark	Strong	<25.0

**Table 7:** Color standards used in color determination of amber-colored liquids.

Standards	Color Scale	Range	Methods	Applications
**ASTM D 1500**	ASTM D 1500	0.5–8.0	Visual Comparator	Petroleum products, lubricating oil, heating oils and diesel fuel oils
**ASTM D 156**	Saybolt Color	From 30 to −16	Colorimeter	Automobile and aviation gasoline, jet fuel, naphtha, kerosene, petroleum waxes and pharmaceutical white oils
**ASTM D 1544**	Gardner Color	1–18	Comparator	Resins, varnishes, lacquers, drying oils, fatty acids, lecithin, sunflower oil and linseed oil
**Lovibond**	Lovibond RYBN	84 calibrated glass color standards	Visual Colorimeter	Color of oils, chemicals, foods and beverages
**United States Standards for Grades of Extracted Honey**	Pfund Scale	Water white, extra white, white, extra light amber, light amber, amber and dark amber	Visual Comparator	Honey
**Standard Reference Method**	SRM Value	1–70	Spectroscopy	Wort or beer
**European Brewery Convention**	EBC Value	4–140	Spectroscopy	Wort or beer
**United States Standards for Grades of Maple Syrup**	Grade A Classification	Golden, amber, dark and very dark	Spectroscopy	Maple syrup
	CIE Values	*XYZ* tristimulus values *xyY* chromacity coordinates CIE L*a*b* color space (−199 to 270) CIE L*C*h $\Delta E_{ab}{}^{*}$	Colorimeter Spectroscopy Image Analysis	Any color of matter

**Table 8:** Network groups.

ANN Model	Prediction	R	MSE
MISO-1	FFA content	0.5764	0.0552
MISO-2	Red Lovibond color	0.8845	0.0589
MISO-3	Yellow Lovibond color	0.8480	0.0904
MIMO-1	Red and yellow Lovibond color	0.8554	0.1054
MIMO-2	FFA content, red and yellow Lovibond color	0.8926	0.0543

**Table 9:** Color values after refining process of nut oil [62].

Refinery Process	Color Values		
	Lightness (L*)	Redness (a*)	Yellowness (b*)
Crude Oil	40.54^c^	2.01^a^	23.71^b^
Neutralized	36.50^c^	0.67^b^	17.62^b^
Bleached	43.22^a^	−1.69^c^	9.95^c^
Deodorized	40.96^b^	−0.87^c^	2.45^d^
LSD	0.1423	0.2250	0.1560

a, b, c, and d after each value indicates the differences of color within the same column. The same letters indicate least significant difference while different letters indicate there is a significant difference of color.

**Table 10:** Colors of the studied edible oils [64].

	Oils	Tiger Nut	Olive	Maize	Sunflower	Soybean
Parameter						
**Color**	Yellow	35	35	35	35	35
	Red	0.9	3.1	0.8	0.8	4

**Table 11:** Equations for estimating the chromatic coordinates and the chroma C of olive oil, measuring the absorbance at only 480 nm and 670 nm [76].

Equation	R^2^	SEE ^a^	RMSE ^b^
L* = 0.556458(A_480_)^2^ − 2.51145A_480_ + 0.55504(A_670_)^2^ − 8.53016A_670_ + 98.4089	0.914	0.74	0.83
a* = 0.177372(A_480_)^2^ + 2.1363A_480_ + 1.43254(A_670_)^2^ − 0.789231A_670_ − 13.9246	0.881	0.54	0.72
b* = −16.0277(A_480_)^2^ + 79.8932A_480_ − 5.06558(A_670_)^2^ + 3.36169A_670_ + 31.9405	0.992	1.28	1.28
*C* = −15.8439(A_480_)^2^ + 78.9312A_480_ − 5.26784(A_670_)^2^ + 3.56917A_670_ + 33.3927	0.992	1.28	1.29

^a^ Standard error of the estimate. ^b^ Root mean squared error.

**Table 12:** RMSE values based on analysis of the data obtained.

Method	RMSE ^YI^	RMSE ^RI^
Visual	0.597	0.275
Automatic	0.338	0.258

^YI^ Yellow index. ^RI^ Red index.

**Table 13:** Standard colors of walnut oils.

Types of Oils	Color	Index	Standard
English Walnut Oil	Color (51/4 Lovibond Scale)	10Yellow/1.5Red Typical	LVO STANDARD
Walnut Oil	Lovibond (Red)	1.5R Max	Kosher Certification Available
Walnut Oil	Lovibond Tintometer 133, 4 mm	Y 30 max. R 4.0 max	-
Natural Walnut Oil for Cooking, Henan, China (Mainland)	A Clear, Bright Yellow Oil	-	SGS 8024–09–07
Walnut Oil, R. Moldova	Color, GOST 5477–93 (at the Request of the Benefit Or in Case of Dispute)	Yellow, max30 mgI_2_/10cm^2^	GD Nr.434 27.05.2010 TR Edible Vegetable Oils

**Table 14:** Color indexes of stripped and non-stripped linseed oil subjected to microwave heating.

Heating Time (Min)	Color Index	
	Stripped	Non-Stripped Oil
0	140.38 ± 1.75 ^B,b^	373.50 ± 4.67A ^B,a^
1	149.10 ± 2.01 ^A,b^	381.45 ± 5.15 ^A,a^
2	140.99 ± 1.76 ^B,b^	365.24 ± 4.57B ^C,a^
3	138.45 ± 1.87 ^B,b^	363.84 ± 4.91B ^C,a^
4	81.96 ± 1.02 ^C,b^	357.86 ± 4.47 ^C,a^
5	27.75 ± 0.37 ^D,b^	174.62 ± 2.36 ^D,a^

The same capital letter in the same column indicates there is no significant difference. The same small letter in the same row indicates there is no significant difference for each parameter.

**Table 15:** Correlation established between the color, flavonoids, phenolics and antioxidants [90].

	Color	Flavonoids	Phenolics	Antioxidants
**Color**	1			
**Flavonoids**	0.999	1		
**Phenolics**	0.974	0.977	1	
**Antioxidants**	0.608	0.616	0.771	1

A value closer to 1 indicates stronger positive correlation between the parameters.

**Table 16:** Color parameters of Polish unifloral honeys.

	Black Locust (n = 4)	Goldenrod (n = 2)	Rapeseed (n = 10)	Lime (n = 4)	Heather (n = 3)	Buckwheat (n = 5)
L*						
Average	86.2	83.7	81.8	83.4	67.3	41.5
SD	±1.2	±2.4	±3.3	±3.9	±4.8	±3.1
Median	86.3	83.7	81.7	83.2	69.7	41.8
Min	84.7	82.0	76.5	78.9	61.7	37.3
Max	87.4	85.4	85.8	88.3	70.5	45.6
a*						
Average	−1.6	−1.2	−0.5	−1.1	10.5	31.9
SD	±0.2	±0.7	±0.3	±1.2	±3.5	±1.7
Median	−1.6	−1.2	−0.6	−1.7	9.5	31.8
Min	−1.9	−1.7	−1.2	−1.8	7.6	29.5
Max	−1.4	−0.7	−0.1	0.7	14.3	33.6
b*						
Average	19.6	34.5	28.4	36.3	68.0	69.2
SD	±1.3	±6.3	±6.5	±12.6	±2.3	±4.2
Median	19.4	34.5	29.6	39.1	68.4	69.6
Min	18.2	30.1	16.9	18.6	65.5	63.2
Max	21.4	38.9	38.0	48.3	70.0	74.5

**Table 17:** EBC and color differences of beers.

Beer Category (Sample Number)	EBC Difference	Absolute L*a*b* Difference
Weissbier (30)	0.6	17.4
Weissbier (31)		
Alcohol-free beer-based mixed drink with lemon juice (5)	0.3	12.6
Belgian strong pale ale (28)		
Alcohol-free beer-based mixed drink with grapefruit juice (6)	0.1	7.7
European pale lager (18)		
International amber lager (24)	1.6	7.3
Irish red ale (34)		
Irish stout (35)	1.1	4.5
Dunkles bock (36)		

**Table 18:** Semi-refined cotton oil process with its color [110].

Oil State during the Process	Color
Crude oil (C)	
First refining (1R)	
Second refining (2R)	
Washing (W)	
Washing and drying (WD)	
Final product (semi-refined oil) (FP)	

**Table 19:** Average accuracy according to the color classifier and model.

	Color Model	KNN	QDA	ELM
Average Accuracy (%)	RGB	90.6	94.7	95.8
	YIQ	92.7	94.7	95.8

**Table 20:** Calibration model results.

Model	LV	RMSEC	RMSECV	R2	Slope	Bias	Correlation
RGB	6	2.60	4.73	0.97	0.97	7.15 × 10^−7^	0.98
HSB	4	3.14	4.33	0.97	0.97	5.88 × 10^−7^	0.98
Grayscale	4	2.06	3.63	0.98	0.98	−1.28 × 10^−6^	0.99

LV = latent variables.

**Table 21:** Analysis of methods for color determination of amber-colored liquids.

	Methods	Equipment	Sample Preparation	Destructive	Wavelength (nm)	Model Equation	AI-Based Processing	Accuracy	Ref No.
**Mineral Oil**	Visual Examination	✓	✕	✕	NA	NA	NA	NA	[23]
	Visual Comparator	✓	✕	✕	NA	✕	NA	NA	[25]
		✓	✕	✕	NA	✕	NA	NA	[45]
	UV-Vis Spectroscopy	✓	✕	✕	400–700	✓	✕	RMSE = 0.6274	[62]
		✓	✕	✕	400–700	✓	✕	R^2^ = 0.99971	[67]
		✓	✓	✕	300–800	✓	✕	RMSEP = 0.18, 0.23	[68]
		✓	✓	✕	300–700	✓	✕	NA	[74]
		✓	✕	✕	590, 840	✓	✕	R^2^ = 0.9974	[72]
	Image Analysis	✓	✕	✕	NA	✕	✕	NA	[100]
**Olive Oil**	UV-Vis Spectroscopy	✓	✕	✕	300–800	✕	✕	NA	[77]
		✓	✓	✕	380–770	✕	✕	NA	[75]
		✓	✕	✕	380–770	✓	✕	color diff. = 3.6	[79]
		✓	✕	✕	430–480 and 660–670	✓	✕	R^2^ = 0.881–0.992	[76]
	Image Analysis	✓	✓	✓	NA	✕	✕	AAE = 3%	[113]
**Palm Oil**	Visual Colorimeter	✓	✕	✓	NA	✕	✓	MSE = 7.0348 × 10^−5^	[53]
	UV-Vis Spectroscopy	✓	✓	✓	RGB	✓	✕	R^2^ = 0.9236	[80]
		✓	✓	✓	380–780	✕	✕	NA	[84]
	Image Analysis	✓	✓	✓	NA	✕	✓	MSE = 0.0011	[81]
**Nut Oil**	Automatic Colorimeter	✓	✓	✕	NA	✕	✕	NA	[62]
		✓	✓	✕	NA	✕	✕	NA	[63]
		✓	✓	✕	NA	✕	✕	NA	[64]
	UV-Vis Spectroscopy	✓	✓	✕	400–700	✓	✕	R^2^ = 0.8717, 0.854	[21]
		✓	✓	✕	400–700 Abs–460, 550, 620, 670	✓	✕	R^2^ = 0.8717 @ 20 °C, R^2^ = 0.854 @ 40 °C	[87]
		✓	✓	✕	400–700	✕	✕	NA	[86]
**Maple Syrup**	UV-Vis Spectroscopy	✓	✓	✕	Abs = 560	✕	✕	NA	[94]
		✓	✓	✕	Abs = 560	✕	✕	NA	[20]
		✓	✓	✕	Abs = 560	✕	✕	NA	[95]
	Fluorescence Spectroscopy	✓	✓	✓	230–370	✓	✕	R^2^ = 0.91 and 0.88	[101]
		✓	✓	✕	Abs = 560	✕	✕	NA	[104]
**Honey**	Automatic Colorimeter	✓	✕	✕	NA	✕	✕	NA	[22]
	UV-Vis Spectroscopy	✓	✓	✕	380–780	✕	✕	NA	[93]
		✓	✓	✕	450–720	✕	✕	NA	[91]
		✓	✓	✕	ABS = 450, 635, 720	✕	✕	NA	[90]
	Image Analysis	✓	✕	✕	NA	✕	✓	NA	[106]
		✓	✓	✕	NA	✕	✓	Accuracy ≥ 90%	[108]
		✓	✕	✕	NA	✓	✓	R^2^ = 0.97	[36]
**Beer**	UV-Vis Spectroscopy	✓	✕	✕	450–800,	✕	✕	NA	[97]
					900–1200				
		✓	✓	✕	380–780 Abs = 430	✕	✕	NA	[99]
		✓	✓	✕	430	✓	✕	R^2^_Vlow_ = 0.9677, R^2^_VHigh_ = 0.9963	[98]
	Image Analysis	✓	✕	✕	NA	✓	✕	NA	[112]
		✓	✕	✕	NA	✓	✓	100%	[111]

NA = not applicable. ✕ = not needed or not applied, ✓ = needed or applicable.
